# A Systematic Review of the Effect of Post-traumatic Stress Disorder Programs for Nurses

**DOI:** 10.1192/j.eurpsy.2023.2080

**Published:** 2023-07-19

**Authors:** I. Yoo, J. Park, S. Choi, Y. Kim, H. Lee

**Affiliations:** 1 Department of Education, Konkuk University; 2 Mo-Im Kim Nursing Research and College of Nursing; 3 College of Nursing and Brain Korea 21 FOUR Project, Yonsei University, Seoul, Korea, Republic Of

## Abstract

**Introduction:**

Nurses are at an increased risk for work-related stress resulting in post-traumatic stress disorder (PTSD). They are susceptible due to frequent exposure to traumatic situations providing care for patients.

**Objectives:**

The purpose of this systematic review is to comprehensively review the content and characteristics of intervention programs for reducing the post-traumatic stress of nurses or nursing students, providing a basis for developing a standardized protocol for programs to promote the integrated health of nurses and protect them from stress events in clinical environments.

**Methods:**

This is a systematic review. Participants (P) targeted nurses or nursing students; Intervention (I) included intervention programs for reducing post-traumatic stress; Comparison (C) was control groups provided with usual or no interventions ; and Outcomes (O) referred to changes in physical or emotional reactions toward post-traumatic stress. Two researchers searched four databases including PubMed, CINAHL, PsycINFO, and EMBASE with keywords such as “nurse,” “post-trumatic stress disorder,” “program,” and “intervention”. A total of 7,523 studies were searched and 10 studies were included for final analysis (Image 1). The Risk of Bias2 (Image 2) and the Risk of Bias for Non-randomized Study I (Image 3) were used to evaluate the quality the included studies.

**Results:**

The number of studies is increasing, with four studies published before 2020, and six studies published since, of which three in 2022. Definitions of trauma situations to which nurses are exposed included diverse elements such as patient death, workplace violence, the COVID-19 pandemic, and complex trauma experiences due to working environments. Most studies have provided multiple intervention sessions, which is appropriate considering the characteristics of PTSD. Most studies examined the secondary effects on mental health such as anxiety, depression, and burnout caused by stress rather than evaluating stress itself. The quality of the study was generally highly biased. The risk of bias increased for the two randomized controlled trials in terms of measurement outcomes and outcome description. The other eight non-randomized studies all included a self-reporting questionnaire of participants, leading to a risk of bias in terms of measurement outcomes.

**Image:**

**Figure:**
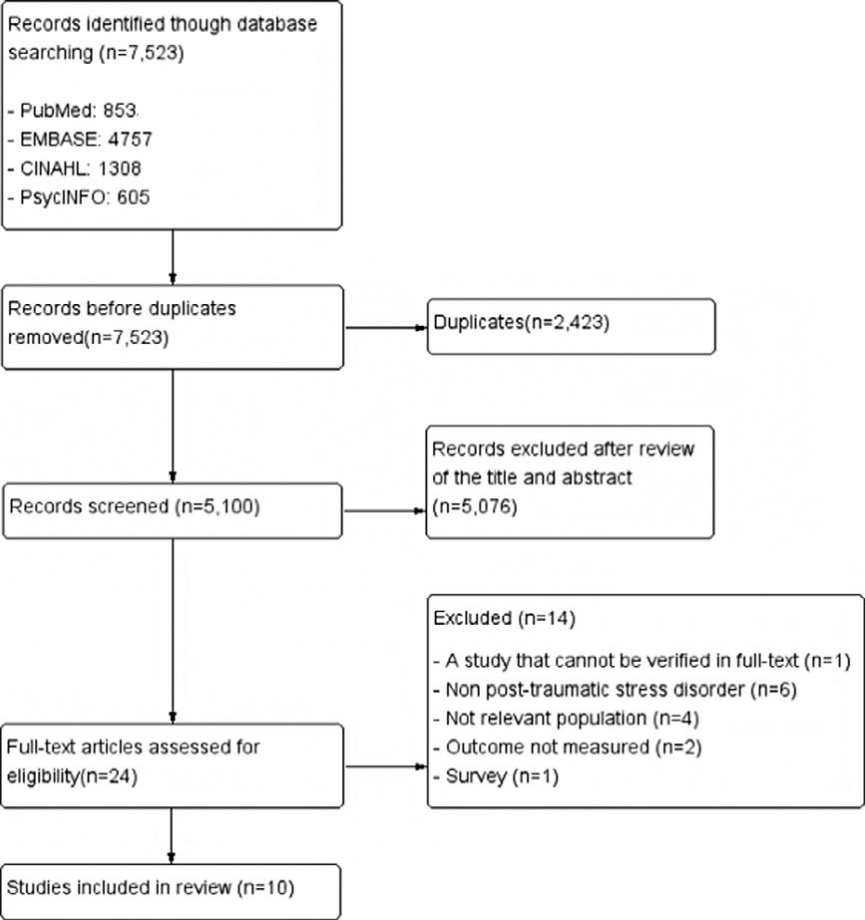

**Image 2:**

**Figure:**
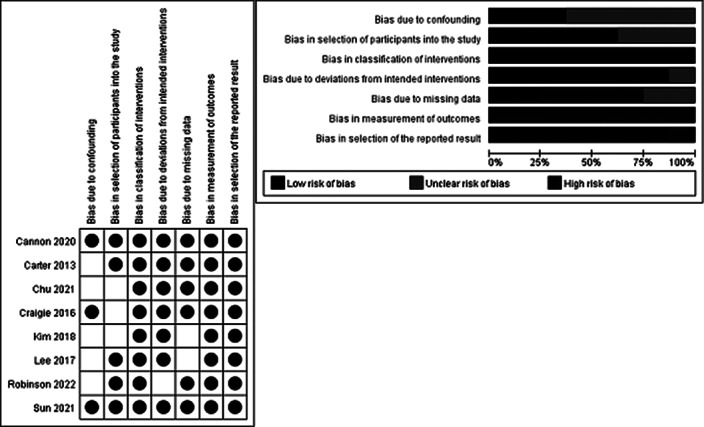

**Image 3:**

**Figure:**
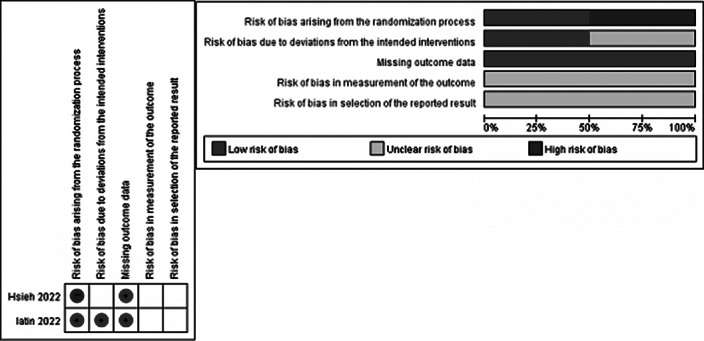

**Conclusions:**

Studies have been conducted to confirm the effectiveness of interventions given heightened concerns about PTSD in nurses. However, the concept of the trauma experienced by nurses was not integrally defined, and information on interventions was often limited. Efforts are required to improve the quality of research in terms of experimental study design.

**Disclosure of Interest:**

None Declared

